# Rifampicin versus streptomycin for brucellosis treatment in humans: A meta-analysis of randomized controlled trials

**DOI:** 10.1371/journal.pone.0191993

**Published:** 2018-02-20

**Authors:** Fanjie Meng, Xiangpo Pan, Wenzhen Tong

**Affiliations:** 1 Yidu Central Hospital of Weifang, Clinical Laboratory, Qingzhou, Shandong, China; 2 Weifang People's Hospital, Weifang, Shandong, China; Universita degli Studi di Firenze, ITALY

## Abstract

Brucellosis is a zoonotic disease with a high morbidity in developing countries, but there the optimal treatment is not yet determined. Therefore, the development of a simple and effective treatment is important. The aim of this study was to summarize the available evidences and compare rifampicin with streptomycin in human brucellosis with doxycycline as background regimen. We systematically searched PubMed, EmBase, and the Cochrane Library from their inception up through December 2016. We included studies with a randomized controlled design that evaluated the effect of streptomycin compared with rifampicin in human brucellosis patients who received doxycycline therapy as background regimen. The overall failure and relapse were summarized using random-effects model. Our meta-analysis included 1,383 patients with brucellosis from 14 trials. We found that patients who received rifampicin therapy had a higher risk of overall failure (RR: 2.36; 95% CI: 1.72–3.23; P<0.001) and relapse (RR: 2.74; 95% CI: 1.80–4.19; P<0.001) compared with streptomycin. Results of the sensitivity analysis were consistent with the overall analysis. Subgroup analysis indicated that mean age of the patients and percentage of male participants might influence the treatment effects. Furthermore, no publication bias was detected. The findings of this study indicated that rifampicin therapy significantly increased the risk of overall failure and relapse compared with streptomycin. Hence, it can be recommended to patients with human brucellosis receiving streptomycin therapy.

## Introduction

Brucellosis is a systemic infection caused by facultative intracellular bacteria of the genus *Brucella* and manifests as fever of unknown origin. It remains a critical public health issue in the Mediterranean region and other developing countries [1]. Moreover, brucellosis is an occupational disease in developed countries, and is contacted by ingestion of contaminated foods imported from other parts of the world [2–4]. For many years, the most commonly used antibiotics for treatment of brucellosis were tetracycline, trimethoprim-sulfamethoxazole, aminoglycosides, rifampicin, quinolones, chloramphenicol, doxycycline, and streptomycin [5–7]. Currently, the standard treatment regimens for brucellosis include a combination of the above antibiotics owing to its higher incidence of failure or relapse in patients who received monotherapy [7].

According to the findings of Food and Agriculture Organization-WHO Expert Committee on Brucellosis in 1986, combination of oral rifampin (600–900 mg/day) and doxycycline (200 mg/day) for 6 weeks was the treatment of choice in patients with acute brucellosis [8]. Doxycycline has already been widely used in the treatment of brucellosis owing to its longer half-life and fewer side effects. But doxycycline combined with streptomycin might induce a higher cure rate and lower relapse rate.

Several trials have indicated that rifampicin therapy may increase the risk of overall failure [9–12], whereas the results of 10 other randomized controlled trials (RCTs) showed no significant difference between rifampicin and streptomycin for overall failure [13–22]. Clarification regarding the optimal treatment strategies in patients with brucellosis is particularly important, as this has not been definitively determined. Therefore, we attempted a large-scale examination of the available RCTs to determine the comparison of rifampicin and streptomycin in patients with brucellosis. Furthermore, we explored if the treatment effects differed in patients with different characteristics.

## Methods

### Data sources, search strategy, and selection criteria

This review was conducted and reported according to the Preferred Reporting Items for Systematic Reviews and Meta-Analysis Statement issued in 2009 (S1 Checklist) [23].

Studies with a randomized controlled design that evaluated the effect of rifampicin compared with streptomycin in patients with brucellosis were eligible for inclusion in this meta-analysis, with no restrictions on language or publication status. Three electronic databases (PubMed, EmBase, and Cochrane Library) were searched for published studies from database inception through December 2016. The search terms used were ("brucella" OR "brucellosis" OR "human brucellosis") AND ("treatment" OR "therapy") AND "clinical trial." The details of search terms for each database are listed in S1 Table. Similar terms were used to search http://www.ClinicalTrials.gov for ongoing RCTs, which had been registered as completed but were not yet published. Finally, manual searches of reference lists from all the relevant studies were conducted to identify any potential eligible studies. The study topic, design, disease status, intervention, control, and outcomes reported were used to identify the potential included studies.

A study was eligible for inclusion if the following criteria were met: randomized controlled design; patients with human brucellosis; patients received rifampicin or streptomycin; and the study that reported the incidence of overall failure or relapse. The exclusion criteria included were as follows: repeated literature; literature that contained unrelated or missing information; study of brucellosis in animals; literature that did not report any desirable outcomes; or study on human tissues.

### Data collection and quality assessment

Data collection and quality assessment were independently performed by two reviewers. Any inconsistencies were adjudicated by an additional reviewer with reference to the original studies. The collected data included first author’s name, publication year, country, sample size, mean age, percentage of male participants, intervention, and control. The outcome variables included overall failure and relapse. One author entered the retrieved information into the computer, while the other author checked it. The Cochrane Collaboration’s tool was employed to evaluate the methodological quality due to its fairly comprehensive nature and has partially validated the quality evaluation of RCTs in meta-analysis [24]. It is based on the following six subscales: sequence generation, allocation sequence concealment, blinding, incomplete outcome data, selective outcome reporting, and other potential sources of bias. The summary risk for bias was defined as low, high, or unclear.

### Statistical analysis

The effect of rifampicin on the risk of overall failure and relapse compared with streptomycin was evaluated based on the occurred events and total patients in each group in an individual study. The random-effects model was employed to calculate the relative risks (RRs) and 95% confidence intervals (CIs) for rifampicin versus streptomycin for the treatment of human brucellosis [25,26]. The heterogeneity among trials was investigated by using the Q statistic and p<0.10 was regarded as an indication of significant heterogeneity [27,28]. Sensitivity analysis was conducted to illustrate the influence of a single study on the meta-analysis [29]. Subgroup analyses were conducted for overall failure and relapse on the basis of publication year, country, sample size, mean age, and percentage male. P values for heterogeneity between subgroups were evaluated by Chi-square test [30]. Funnel plots, Egger [31] and Begg [32] tests, were employed to evaluate the publication bias for overall failure and relapse. P values for the summary results are 2-sided and p<0.05 was considered statistically significant. All statistical analyses were performed using STATA software (version 12.0; Stata Corporation, College Station, TX, USA) and the study quality assessment using Review Manager, version 5.3 (Cochrane Collaboration, Oxford, England).

## Results

**Fig 1:**
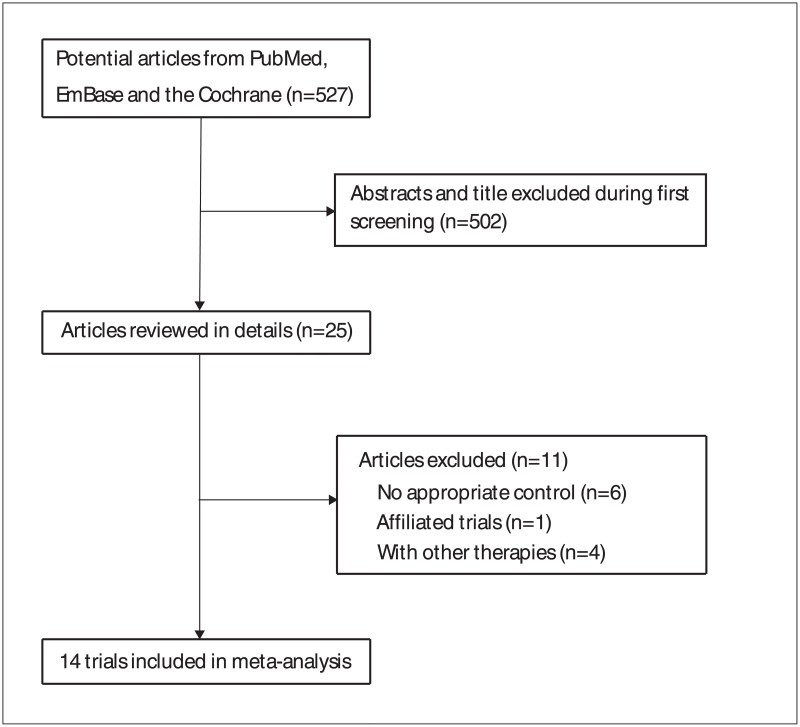
Study selection process.

**Fig 2:**
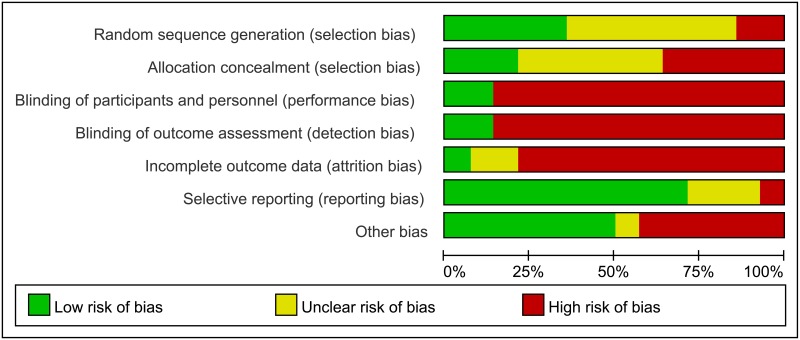
Risk of bias graph of included RCTs.

**Fig 3:**
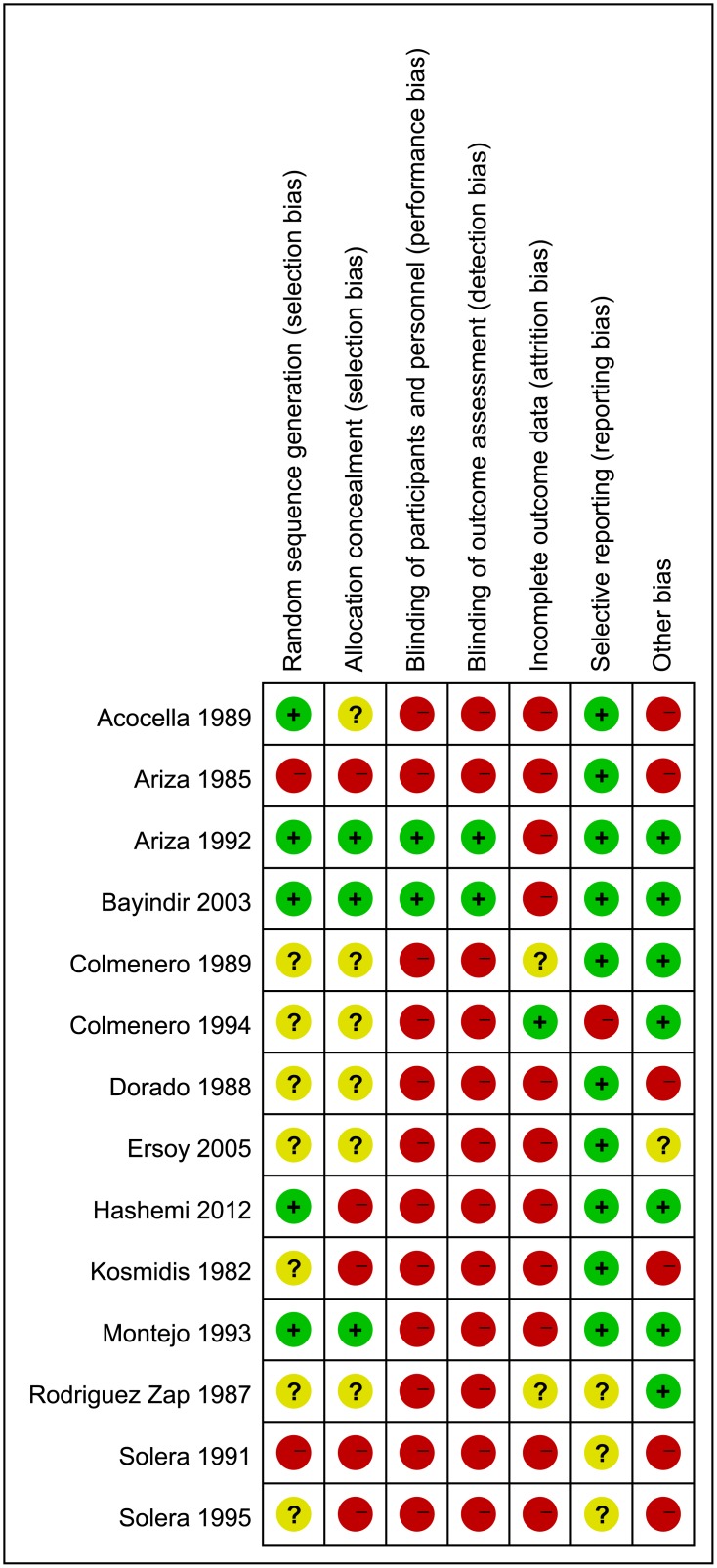
Risk of bias summary of included RCTs.

The primary search retrieved 527 articles. After scanning the titles and abstracts, 502 irrelevant or duplicate articles were excluded. Of the total, 25 potentially eligible studies were retrieved, and 14 RCTs met the inclusion criteria of this meta-analysis [9–22]. Results of the study-selection process are shown in Fig 1. The search of http://www.ClinicalTrials.gov and the reference lists of potentially relevant studies yielded no studies that met the inclusion criteria. Table 1 summarized the general characteristics of the included studies. The sample size ranged from 19 to 194, with a total of 1,383 patients with human brucellosis. Eleven trials were conducted in Europe and the remaining three trials in Asia. The mean or median age ranged from 26.4 to 46.0 years and the percentage of male participants ranged from 37.0 to 82.0%. All patients in the included trials received doxycycline as background therapy. The quality assessment of 14 included trials was conducted using the Cochrane Collaboration tool, and the results of quality analysis are presented in Figs 2 and 3.

**Table 1:** Baseline characteristics of included patients and quality assessment.

Study	Publication years	Country	Sample size	Mean age (years)	Percentage male (%)	Intervention	Control
Acocella [13]	1989	France, Greece, Spain	146	43.0	58.8	Doxycycline 1× 200 mg for 45 days + rifampicin 1× 900 mg for 45 days	Doxycycline 1× 200 mg for 45 days + streptomycin 1×1 g for 21 days
Ariza [9]	1985	Spain	56	33.0	80.0	Doxycycline 1×100 mg for 30 days (45 days)+rifampicin 15 mg/kg/day for 30 days (45 days)	Doxycycline 1×100 mg or tetracycline hydrochloride 4×0.5g for 30 days (45 days)+streptomycin 1×1 g for 21 days
Ariza [14]	1992	Spain	111	26.4	71.6	Doxycycline 2×100 mg for 45 days + rifampicin 15 mg/kg/day for 45 days	Doxycycline 2×100 mg for 45 days+streptomycin 1×1 g for 15 days
Bayindir[15]	2003	Turkey	102	40.5	55.0	Doxycycline 2×100 mg for 45 days + rifampicin 15 mg/kg/day for 45 days	Doxycycline 2×100 mg for 45 days+Streptomycin 1×1 g for 15 days
Colmenero [16]	1989	Spain	111	33.1	69.4	Doxycycline 2×100 mg for 45 days (60 days)+rifampicin 15 mg/kg/day for 45 days (60 days)	Doxycycline 2×100 mg for 30 days (60 days)+Streptomycin 1×1 g for 21 days
Colmenero [17]	1994	Spain	19	33.3	65.0	Doxycycline 2×100 mg for 6 weeks (12 weeks)+rifampicin 10–15 mg/kg/day for 6 weeks (12 weeks)	Doxycycline 2×100mg for 6weeks (12weeks)+Streptomycin 1×1 g for 3 weeks
Dorado[18]	1988	Spain	73	37.2	37.0	Doxycycline 1×200 mg for 28 days + rifampicin 1×1200 mg for 7 days, followed by 1×600 mg for 21 days	Doxycycline 1×200 mg for 40 days+Streptomycin 1×1 g for 21 days
Ersoy[19]	2005	Turkey	129	36.4	52.6	Doxycycline 200 mg/day for 6 weeks + rifampicin 600 mg/day for 6 weeks	Doxycycline 100 mg/day for 6 weeks+ Streptomycin 1 g/day for 3 weeks
Kosmidis[20]	1982	Greece	29	NA	NA	Doxycycline 1×200 mg for 45 days+rifampicin 1×900 mg for 45 days	Doxycycline 1×200 mg for 45 days+Streptomycin 1×1 g for 21 days
Montejo[21]	1993	Spain	130	46.0	74.0	Doxycycline 1×200 mg for 6 weeks + rifampicin 1×900 mg for 6 weeks	Doxycycline 1×200 mg for 6 weeks +Streptomycin 1×1 g for 2 or 3 weeks
Rodriguez Zap[22]	1987	Spain	72	36.0	80.0	Doxycycline 1×200 mg for 45 days + rifampicin 1×900 mg for 45 days	Doxycycline 2×200 mg for 21 days+Streptomycin 1×1 g for 21 days
Solera [10]	1991	Spain	84	32.0	77.0	Doxycycline 2×100 mg for 45 days + rifampicin 1×900 mg for 21 days	Doxycycline 2×100 mg for 45 days+Streptomycin 1×1 g for 14 days
Solera [11]	1995	Spain	194	33.5	82.0	Doxycycline 2×100 mg for 45 days + rifampicin 1×900 mg for 45 days	Doxycycline 2×100 mg for 45 days+Streptomycin 1×1 g for 14 days
Hashemi [12]	2012	Iran	127	39.3	55.1	Doxycycline 1×200 mg for 6 weeks + rifampicin 15 mg/kg/day for 6 weeks	Doxycycline 1×200 mg for 6 weeks + Streptomycin 1×1 g for 3 weeks

**Fig 4:**
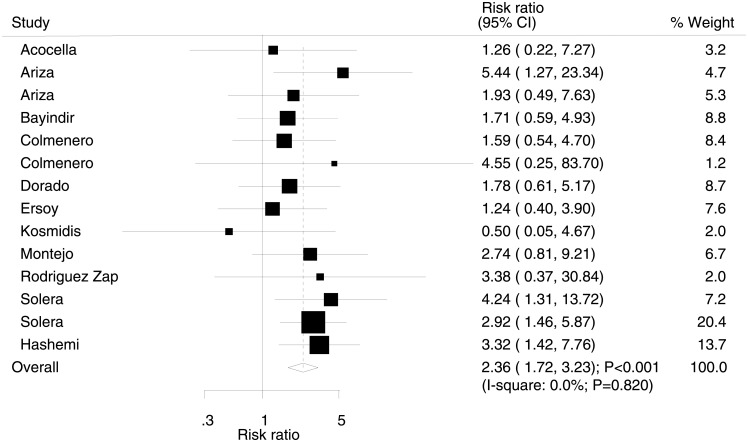
Effect of rifampicin therapy on the incidence of overall failure.

After pooling all the included trials, the summary RR indicated that rifampicin therapy significantly increased the incidence of overall failure compared with streptomycin (RR: 2.36; 95% CI: 1.72–3.23; P<0.001; Fig 4). The heterogeneity test revealed no evidence of heterogeneity (I^2^ = 0.0%, P = 0.820). Sensitivity analysis concluded that the data was not affected by sequential exclusion of individual trials (S1 Fig).

**Fig 5:**
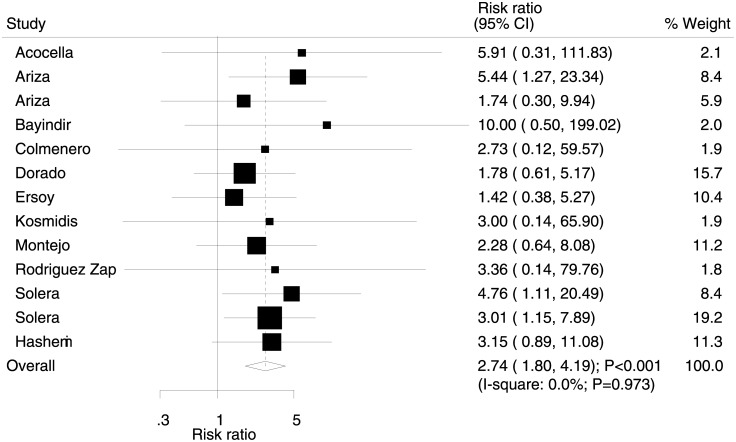
Effect of rifampicin therapy on the incidence of relapse.

Data for the effect of rifampicin on the risk of relapse were available in 13 trials. We noted that rifampicin therapy was associated with higher risk of relapse compared with streptomycin (RR: 2.74; 95% CI: 1.80–4.19; P<0.001; Fig 5), and no evidence of heterogeneity was observed (I^2^ = 0.0%, P = 0.973). Sensitivity analysis was conducted for relapse, and concluded no affect on the data by excluding each specific study (S2 Fig).

Although heterogeneity analysis showed p>0.10 for overall failure and relapse, subgroup analyses were conducted for overall failure and relapse to evaluate the effect of rifampicin in the treatment of human brucellosis within specific subpopulations (Table 2). Overall, we noted that there was no significant difference between rifampicin and streptomycin for overall failure when the mean age of the patients was >40 (RR: 1.92; 95% CI: 0.93–3.97; p = 0.078). Additionally, men with brucellosis who received rifampicin had a higher failure risk than women (ratio of RR: 1.62; 95% CI: 0.85–3.07; p = 0.140). Results of subgroup analyses based on other factors were consistent with the overall analysis.

**Table 2:** Subgroup analysis.

Outcomes	Group	RR and 95%CI	P value	Heterogeneity (%)	P value for heterogeneity	Ratios of RR	P value between subgroups
Overall failure	Publication year						
	After 2000	2.12 (1.18–3.82)	0.012	3.9	0.353	0.86 (0.43–1.73)	0.679
	Before 2000	2.46 (1.69–3.59)	<0.001	0.0	0.807		
	Country						
	Europe	2.46 (1.69–3.59)	<0.001	0.0	0.807	1.16 (0.58–2.33)	0.679
	Asia	2.12 (1.18–3.82)	0.012	3.9	0.353		
	Sample size						
	100 or more	2.39 (1.62–3.51)	<0.001	0.0	0.870	1.04 (0.53–2.02)	0.961
	<100	2.30 (1.34–3.97)	0.003	0.0	0.438		
	Mean age						
	40 or more	1.92 (0.93–3.97)	0.078	0.0	0.742	0.74 (0.33–1.67)	0.473
	<40	2.58 (1.81–3.67)	<0.001	0.0	0.802		
	Percentage male (%)						
	70 or more	3.14 (1.97–4.98)	<0.001	0.0	0.927	1.62 (0.85–3.07)	0.140
	<70	1.94 (1.25–3.01)	0.003	0.0	0.821		
Relapse	Publication year						
	After 2000	2.45 (1.03–5.83)	0.044	0.0	0.432	0.86 (0.32–2.33)	0.770
	Before 2000	2.84 (1.75–4.61)	<0.001	0.0	0.975		
	Country						
	Europe	2.84 (1.75–4.61)	<0.001	0.0	0.975	1.16 (0.43–3.13)	0.770
	Asia	2.45 (1.03–5.83)	0.044	0.0	0.432		
	Sample size						
	100 or more	2.89 (1.61–5.21)	<0.001	0.0	0.926	1.12 (0.48–2.60)	0.798
	<100	2.59 (1.41–4.76)	0.002	0.0	0.807		
	Mean age						
	40 or more	3.15 (1.07–9.31)	0.038	0.0	0.596	1.18 (0.36–3.83)	0.783
	<40	2.67 (1.68–4.25)	<0.001	0.0	0.909		
	Percentage male (%)						
	70 or more	3.16 (1.79–5.60)	<0.001	0.0	0.906	1.39 (0.59–3.27)	0.457
	<70	2.28 (1.20–4.34)	0.012	0.0	0.797		

**Fig 6:**
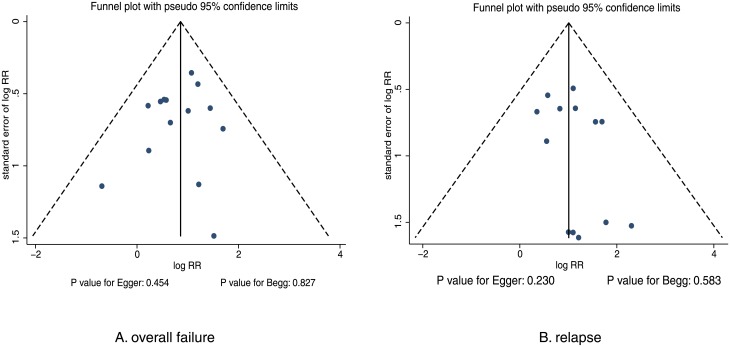
Funnel plots.

A review of the funnel plots could not exclude publication bias for overall failure and relapse (Fig 6). Results of the Egger’s and Begg’s tests showed no evidence of publication bias for overall failure (p = 0.454 for Egger; p = 0.827 for Begg) and relapse (p = 0.230 for Egger; p = 0.583 for Begg).

## Discussion

The current meta-analysis investigated RCTs that compared rifampicin and streptomycin for the treatment of human brucellosis. This comprehensive quantitative study included 1,383 patients with brucellosis from 14 RCTs with a broad range of characteristics. The findings of this study indicated that the incidence of overall failure and relapse was significantly increased for rifampicin over streptomycin. However, a significant difference was not observed if the mean age of the patients was greater than 40 years.

A previous meta-analysis suggested that doxycycline-rifampicin was associated with higher risk of overall failure and relapse when compared with doxycycline-streptomycin for patients with bacteremia or complicated brucellosis. Further, the triple regimen of doxycycline, rifampicin, and aminoglycoside was superior to doxycycline-streptomycin in the treatment of brucellosis. These findings reported no differences between gentamicin and streptomycin and doxycycline plus rifampicin or streptomycin was superior to quinolones plus rifampicin. Finally, they indicated that triple or dual drug regimens were associated with a lower risk of failure than monotherapy when administered for a similar duration [33]. Although this study provided comprehensive comparisons of different treatment regimens, the effect of doxycycline that was available in one of the trial arms and the pooled analyses according to the patients’ characteristics were not calculated. Our meta-analysis also found that the combination of doxycycline with streptomycin was superior to doxycycline plus rifampicin. In addition, there was no significant difference between quinolones plus rifampicin and doxycycline plus rifampicin. These results indicated that intramuscular injection, access to care, and cost should be considered to make a decision when prescribing these regimens [34]. However, no evaluation was conducted on how the treatment effects differed in patients with different characteristics. Solís García del Pozo suggested favorable outcomes of doxycycline-streptomycin over doxycycline-rifampicin, while no significant differences between doxycycline-streptomycin and doxycycline-gentamicin were observed [35]. Although similar conclusions were found in our meta-analysis, the treatment in our study or patients with specific characteristics was conducted, and the comparisons of treatment effects between the subgroups were calculated. Furthermore, the current study included several additional studies and provided more stable outcomes with high statistical power.

Although significant differences were observed, the methodological evaluation of individual trials was limited, as only five trials provided clear information on randomization, blinding, withdrawals and dropouts, and used intention-to-treat analysis. Most of the included trials were of low study quality, which might contribute to the unreliability of the results. Consequently, considering the unsatisfactory quality of majority of the included trials, the findings of this study should be cautiously recommended for the treatment of patients with brucellosis.

The pooled data showed a significantly increased risk of overall failure and relapse in patients who received rifampicin plus doxycycline compared with streptomycin plus doxycycline. Several included trials reported consistent conclusions. Ariza et al. conducted a trial based on 56 patients with human brucellosis to compare the efficacy of rifampicin-doxycycline with streptomycin-doxycycline. Results revealed that rifampicin-doxycycline was less effective in the prevention of relapse than streptomycin-doxycycline when both were administered for 30 days. After which, they recommended a longer period of treatment of rifampicin-doxycycline to yield a lower relapse rate [9]. Furthermore, Solera et al. suggested doxycycline-rifampicin therapy was less effective than doxycycline-streptomycin in patients with acute brucellosis in terms of therapeutic efficacy and relapse [10,11]. In addition, Hashemi et al. recommended doxycycline-streptomycin as a first-line therapeutic regimen and combined doxycycline-streptomycin and ofloxacin-rifampicin as the second-line treatment regimen for the treatment of brucellosis [12]. However, in most other trials, no significant differences were detected for overall failure and relapse. A possible explanation for this could be lower than expected event rate and smaller sample size, which led to broad confidence intervals, i.e., no statistically significant differences.

Subgroup analysis suggested no significant difference between these two regimens if the patient’s mean age was >40 years, which might be due to that the effect of treatment in older patients was diminished compared with younger patients. Although no statistically significant differences between the subgroups were observed, several trends were identified and should be considered and verified in a further large-scale RCT. These conclusions may vary as smaller trials were included in each subset and should be verified further in future studies. We therefore provided a relative result and a comprehensive review.

Several limitations of this meta-analysis were acknowledged. (1) The study comprised mostly trials with a low study quality and the results might vary. (2) Data were taken from only published studies and publication bias may be inevitable. (3) Summary analysis used pooled data as individual data were not available, which restricted a more detailed analysis.

In conclusion, rifampicin was associated with higher incidence of overall failure and relapse when compared with streptomycin in patients with brucellosis. Furthermore, age and gender might influence the treatment effect. We strongly recommend that the interaction of these two factors should be clarified and explore more effective treatment regimens in future studies.

## Supporting information

S1 Checklist PRISMA 2009 checklist. (DOC)

S1 Fig Sensitivity analysis for overall failure. (TIF)

S2 Fig Sensitivity analysis for relapse. (TIF)

S1 Table Search strategies. (DOC)
