# Supplementary figures and images for: Rifampicin versus streptomycin for brucellosis treatment in humans: A meta-analysis of randomized controlled trials
Source: PLoS One. 2018 Feb 20;13(2):e0191993. doi: 10.1371/journal.pone.0191993 (PMC5819773; doi:10.1371/journal.pone.0191993)

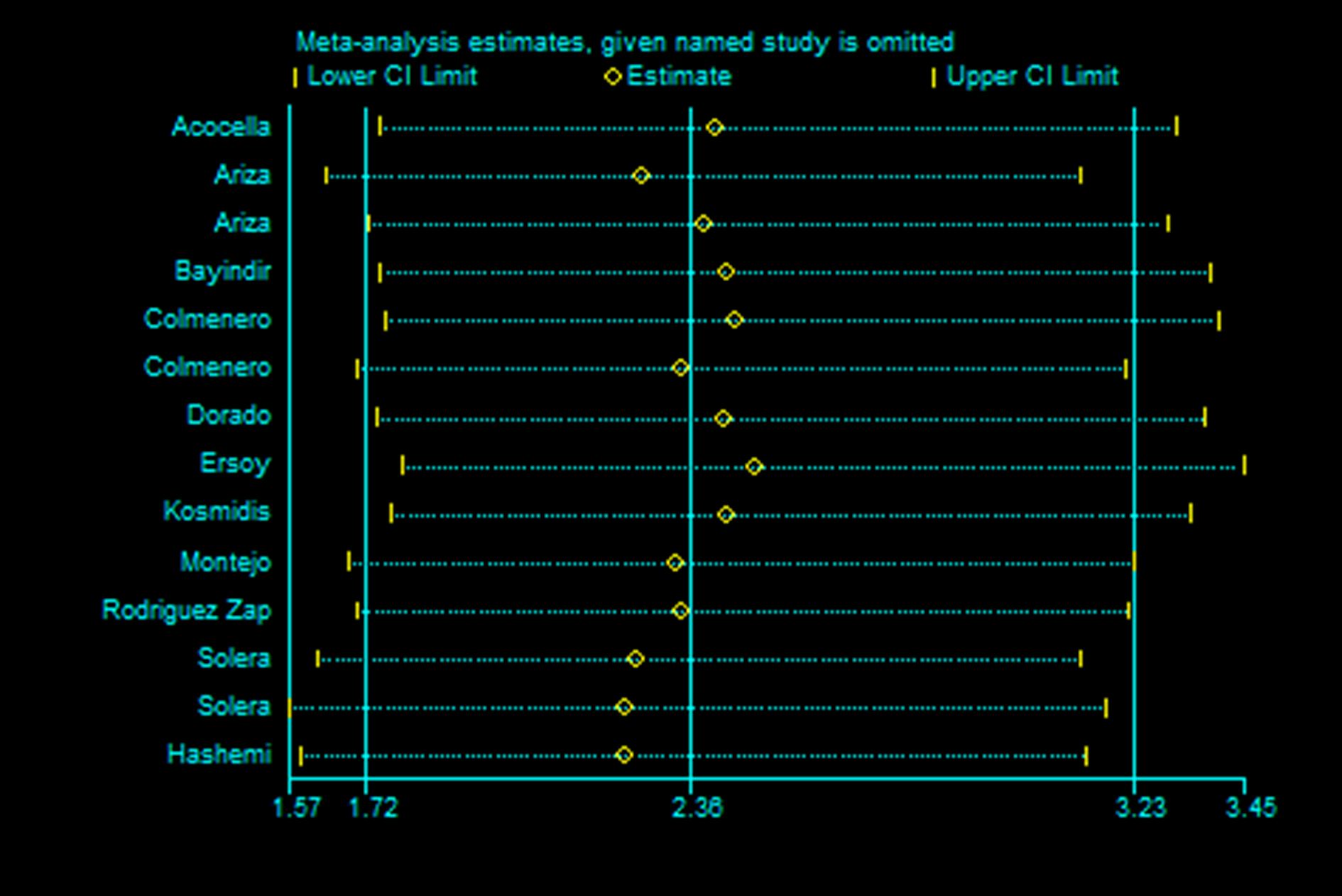

Supplement: S1 Fig — (TIF) [file pone.0191993.s002.tif]

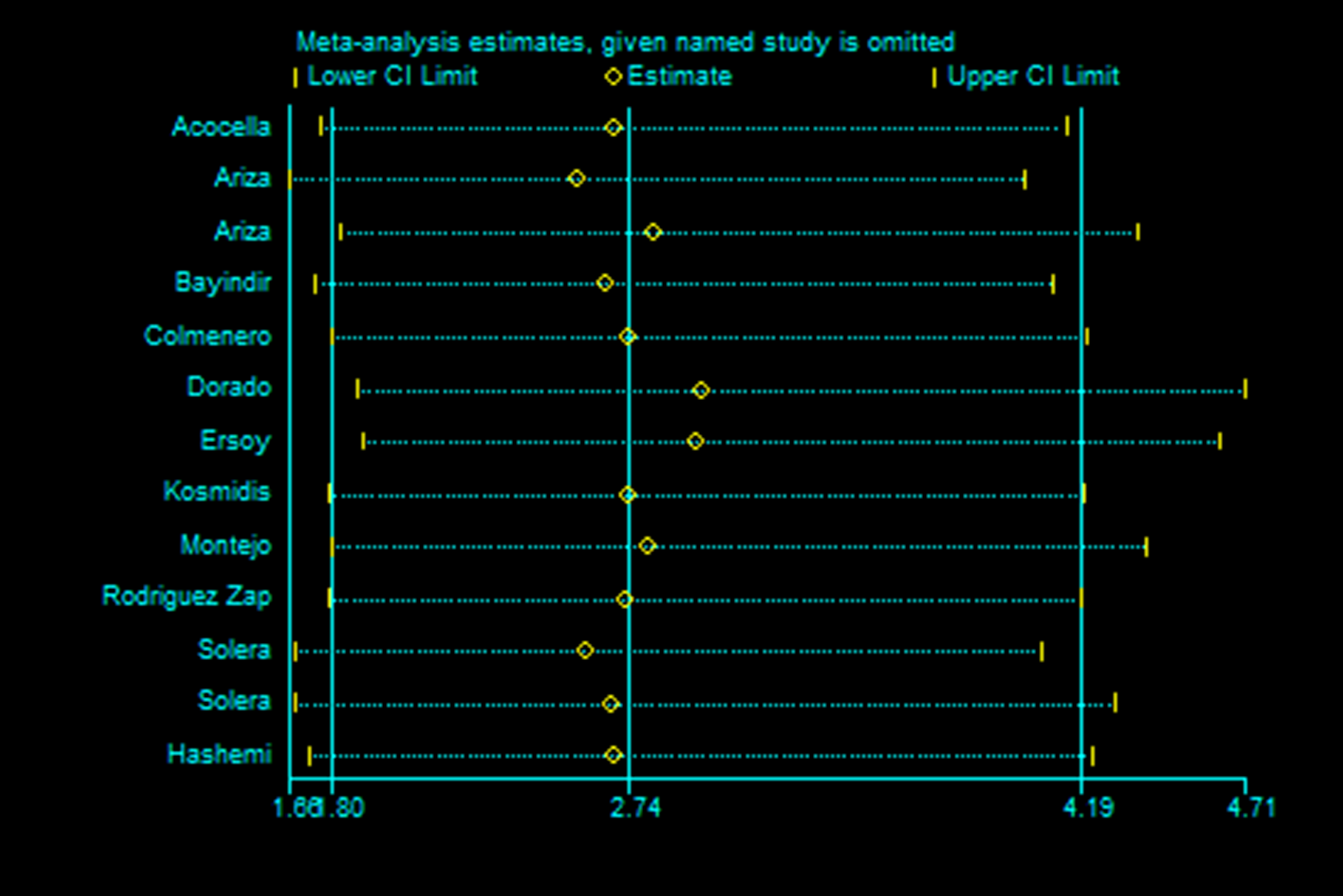

Supplement: S2 Fig — (TIF) [file pone.0191993.s003.tif]
