# Supplementary material for: Rifampicin versus streptomycin for brucellosis treatment in humans: A meta-analysis of randomized controlled trials
Source: PLoS One. 2018 Feb 20;13(2):e0191993. doi: 10.1371/journal.pone.0191993 (PMC5819773; doi:10.1371/journal.pone.0191993)
Supplement: S1 Table — (DOC) [file pone.0191993.s004.doc]

| **Order** | **PUBMED** |
| --- | --- |
| **#1** | "brucella" OR "brucellosis" OR "human brucellosis" |
| **#2** | "treatment" OR "therapy" OR "clinical trial" |
| **#3** | #1 AND #2 |
| **#4** | (#1 AND #3) AND (Clinical Trial[ptyp] AND "humans"[MeSH Terms]) |

| **Order** | **EMBASE** |
| --- | --- |
| **#1** | "human brucellosis" |
| **#2** | "treatment" OR "therapy" OR "clinical trial" |
| **#3** | #1 AND #2 |
| **#4** | #1 AND #2 AND ('clinical article'/de OR 'clinical study'/de OR 'clinical trial'/de OR 'comparative study'/de OR 'controlled clinical trial'/de OR 'controlled study'/de OR 'human'/de OR 'in vitro study'/de OR 'intermethod comparison'/de OR 'major clinical study'/de OR 'prospective study'/de OR 'questionnaire'/de OR 'randomized controlled trial'/de OR 'retrospective study'/de) |

| **Order** | **Cochrane library** |
| --- | --- |
| **#1** | "brucella" OR "brucellosis" OR "human brucellosis" |
| **#2** | "treatment" OR "therapy" AND "clinical trial" |
| **#3** | #1 AND #2 |
